# Multi-Type Node Detection in Network Communities

**DOI:** 10.3390/e21121237

**Published:** 2019-12-17

**Authors:** Chinenye Ezeh, Ren Tao, Li Zhe, Wang Yiqun, Qu Ying

**Affiliations:** 1 Software College, Northeastern University, Shenyang 110000, China; noblenenye@gmail.com (C.E.); gislzneu@163.com (L.Z.); neuwangyiqun@163.com (W.Y.); qybefore1727@163.com (Q.Y.); 2 Department of Computer Engineering, Michael Okpara University of Agriculture, Umudike 440109, Nigeria

**Keywords:** bridging centrality, community detection, disjoint nodes, disjunct nodes, node similarity, overlapping nodes

## Abstract

Patterns of connectivity among nodes on networks can be revealed by community detection algorithms. The great significance of communities in the study of clustering patterns of nodes in different systems has led to the development of various methods for identifying different node types on diverse complex systems. However, most of the existing methods identify only either disjoint nodes or overlapping nodes. Many of these methods rarely identify disjunct nodes, even though they could play significant roles on networks. In this paper, a new method, which distinctly identifies disjoint nodes (node clusters), disjunct nodes (single node partitions) and overlapping nodes (nodes binding overlapping communities), is proposed. The approach, which differs from existing methods, involves iterative computation of bridging centrality to determine nodes with the highest bridging centrality value. Additionally, node similarity is computed between the bridge-node and its neighbours, and the neighbours with the least node similarity values are disconnected. This process is sustained until a stoppage criterion condition is met. Bridging centrality metric and Jaccard similarity coefficient are employed to identify bridge-nodes (nodes at cut points) and the level of similarity between the bridge-nodes and their direct neighbours respectively. Properties that characterise disjunct nodes are equally highlighted. Extensive experiments are conducted with artificial networks and real-world datasets and the results obtained demonstrate efficiency of the proposed method in distinctly detecting and classifying multi-type nodes in network communities. This method can be applied to vast areas such as examination of cell interactions and drug designs, disease control in epidemics, dislodging organised crime gangs and drug courier networks, etc.

## 1. Introduction

Over the years, numerous research works have been devoted to identification and description of community with respect to networks or graphs without a consensus on its definition [1]. Some characteristic features can easily be extracted from the nodes in a graph to describe a community [2,3]. Intuitively, communities are usually acquired from the removal of bridges (edges), bridge-nodes or articulation points (cut vertexes) from a graph. Identification and removal of these sets of nodes and edges can effectively disintegrate a network naturally into densely connected subgroups [4,5,6,7,8,9,10,11]. A community can effectively be described as clusters of densely connected nodes that are revealed along disconnected lines of weak connections of bridge-nodes.

Communities are very useful in detecting hierarchical clusters in various fields such as cells interaction, epidemic/disease control in natural and biological sciences, design of power grid and road networks in engineering, collaboration networks, social networks in social sciences and so on [6,7,11,12,13]. Most networks reveal hierarchical structures, i.e., they reveal smaller clusters contained within larger clusters. One of the most popular clustering methods is the hierarchical clustering method, which is further divided into two categories namely agglomerative algorithms and divisive algorithms. In agglomerative algorithms, clusters of nodes with high similarity are merged together in successive iterations to achieve better clusters, whereas in divisive algorithms, nodes with low similarity values are disconnected in successive iterations to reveal better clusters of nodes with higher similarity [1,14].

In recent years, existing community detection algorithms reported in the literature were specifically designed to either detect only disjoint nodes or overlapping nodes. Disjoint nodes, also known as node clusters, are nonoverlapping groups of densely connected subgraphs of a network [1,12,14,15,16,17,18]. Overlapping nodes are nodes shared by two or more communities at the same time, thereby creating overlapping communities [1,14,15,16,19,20,21,22]. Previous methods rarely take into consideration disjunct nodes (isolated or neutral nodes) [23]. However, when critically examined, real complex networks reveal the existence of multi-type nodes [1]. For example, Peel et al. [24] reported that the majority of community detection algorithms cannot recover the metadata of a certain node or often mislabelled this node (person number 9) in the popular Zachary’s karate club network, which, most likely, had a neutral political support during the feud that eventually divided the karate club. Nodes of this type can only be discovered by suitably designed algorithms that are capable of distinguishing the different node types on a network.

There has been proliferation of different community detection algorithms over the past few years, with each algorithm being designed to achieve what has already been attained in the past with little or no difference. The idea of implementing these algorithms differently on datasets for set purposes not only consume much resources but take quite precious amount of time. We set out to achieve a unified process of community detection which focuses on and reveals the various node types, and therefore we propose a method that detects multi-type nodes in network communities that disintegrate a network into communities. This method ensures that various node types are recovered and duly classified. In other words, when an overlapping node is identified, it is easier to distinguished the communities been overlapped by it. Also, the disjoint nodes are clearly separated whereas the disjunct nodes do not adhere to any clusters. Some of the foremost community detection algorithms were proposed by Girvan and Newman [4,5]. In these algorithms, the edge with the highest betweeness centrality value is iteratively disconnected until the network disintegrates into modules. It is reported that these algorithms cannot discover overlapping nodes, as each node is assigned to a cluster [1]. However, we know that most real networks often share nodes between communities, resulting in community overlap and sometimes disjunct nodes are discovered [1,22]. In their work [5], Newman and Girvan introduced a quality measure known as modularity measure, which is used to determine the strength of community structures found by the algorithm. This measure further inspired other community detection algorithms based on modularity optimisation methods. Newman [25] proposed a fast optimisation of the quality function modularity. In this method, at the initial stage, there are |N| communities formed by each node. At every successive iteration, communities are merged only if it improves the value of the quality function modularity [1,25]). Even though Newman’s method is quite fast and detected quality communities on networks, Clauset et al. [26] pointed out that it consumed much storage space and time in the computation of adjacency matrix. As a result, they proposed a more efficient method known as greedy modularity optimisation algorithm, which uses data structures to compute and retain only significant improvements in the value of the quality function modularity [1,26]). Similar to the greedy modularity optimisation techniques of Newman [25] and Clauset et al. [26] is the very popular Louvain algorithm [27]. This method is suitable for both weighted and unweighted networks. In the first phase, each node is assigned to its own community. Nodes are joined to form supernodes only if there is gain in the value of modularity. The second phase involves fusion of connected supernodes on the condition that the value of modularity increased. The entire process is repeated recursively until gain in the value of modularity is no longer possible. The Louvain algorithm is reported to be one of the fastest community detection algorithms and is capable of handling networks with millions of nodes and edges [1]. The modularity optimisation methods fall under the category of hierarchical agglomeration community detection algorithms, and they detect only disjoint or none overlapping clusters. Unlike the modularity optimisation based methods, Label propagation algorithm (LPA) proposed by Raghavan et al. [28] uses only structural information of networks to detect communities. At the initial stage, each node obtains a unique identifier or label and subsequently adopts the majority label of its neighbours after every successive propagation iteration. The propagation process terminates when a convergence point is reached, i.e., when every node adopts the majority label of its neighbours or the preassigned number of iterations is attained. At this stage, densely connected clusters of nodes assume same label thereby forming communities [1,28]. The Spectral algorithm is a matrix-based clustering method that uses eigenvectors for clustering. Here, the nodes on a network form data points and the edges between nodes form distances. The eigenvector of these points is calculated from the generated affinity matrix, and a clustering method such as the *k*-means clustering technique is used to partition these points [1,29,30]. As noted earlier, complex networks have the tendency to allow multi-membership of nodes to two or more communities per time and, consequently, this brings about node overlaps and overlapping communities in networks [22,31,32]. To capture such distinctive characteristics of networks, researchers proposed and designed community detection algorithms that are capable of capturing the overlapping structures of complex networks. Yuan et al. [19] proposed a constraint model that necessitates recursive edge-cuts that meet the constraint condition. This algorithm detects overlapped communities at the end of the process.

Note that the majority of the previously proposed algorithms can only detect disjoint nodes (node clusters) or overlapping nodes (nodes binding overlapping communities) and rarely disjunct/neutral nodes (single node partitions). We propose a new method which distinctly identifies disjoint nodes, disjunct nodes and overlapping nodes following a natural pattern of network division. Our approach rather focuses on identifying the various node types, as when these node types are identified, network communities are naturally recovered. The procedure involves iteratively finding nodes with the highest bridging centrality value and subsequently its neighbours that yield the least node similarity value are determined and the links joining them disconnected [33]. The process is sustained until a stoppage criterion condition is met. Our approach focuses on revealing the node types and this ensures that nodes are distinctly identified as well as classified into communities with high value of modularity. Singleton nodes with a degree value of one are ignored to avoid the possibility of cutting them off during network division, so as not to mix them up with what we classify as disjunct nodes in this work. Additionally, the properties that characterise disjunct/neutral nodes are highlighted and clearly demonstrated. The proposed algorithm was tested and compared with other community detection algorithms on artificial and real-world datasets, and the results indicated impressive performance against the compared algorithms.

The outline for the rest of this paper is as follows. In Section 2, we define some relevant terms and design and implement an algorithm to detect disjoint nodes, disjunct nodes and overlapping nodes. We further highlight some of the properties that characterise disjunct nodes. We analyse the experimental results, discuss our findings and offer recommendations in Section 3. Finally, we conclude in Section 4.

## 2. Methodology

Bearing in mind the usefulness of communities in studying and understanding patterns of node connectivity on networks, we propose a new method to discover disjoint nodes, disjunct nodes and overlapping nodes. Our method iteratively identifies bridge-nodes using the Bridging centrality metric [6] to compute the nodes with the highest bridging centrality value. Furthermore, the node similarity value between the identified bridge-node and all of its neighbours is calculated. We rank the node similarity values in decreasing order and detach the edges/links with the least node similarity value. Intuitively, the bridge-node forms a community by aligning with its neighbours that return high node similarity values unless there is anything to the contrary [3,34]. The edge/link which has the least node similarity value is the edge between the bridge-node and another community. If the node similarity values between the bridge-node and its neighbours return a value equal to zero, then the bridge-node would most certainly be isolated upon network division and we classify this node to be a disjunct node without any community. This signifies that the isolated nodes do not share any nodes in common with any of their neighbours. Some of the bridge-nodes which seem to be isolated are actually overlapping nodes. The proposed algorithm identifies them by cutting them out just like the isolated nodes, but they differ from isolated nodes in the sense that they have paths linking back to them from their neighbours, they share some common nodes and can form communities with their neighbours.

The proposed algorithm is designed to be implemented on a typical undirected and unweighted graph G=(V,E), in which $V=\left\{v_{1},v_{2}\cdots v_{n}\right\}$ is of *n* nodes and $E=\left\{e_{1},e_{2}\cdots e_{m}\right\}$ is a set of edges denoted by *m*. The *n* nodes and their connections are represented by an adjacency matrix $=\left[A_{ij}\right]\left(n\times n\right)$ where $A_{ij}=1$ if $v_{i}$ is connected to $v_{j}$, and $A_{ij}=0$ otherwise.

### 2.1. Definition of Important Measures and Terms

#### 2.1.1. Similarity Measure

The node similarity measure is used to compute the level of relationship between nodes. This measure is equally used to ascertain if nodes can be grouped together into the same community [1,3,16]. We determine the similarity between nodes via the structural similarity, which computes the intersections between the neighbourhood sets of any two nodes. There are a couple of node similarity measures but we adopt the Jaccard similarity coefficient because of its intuitive appeal. The model is shown in Equation (1).

(1) $$\frac{|n_{i}\cap n_{j}|}{|n_{i}\cup n_{j}|}\;$$

$n_{i}$ is the neighbourhood set of node *i* and $n_{j}$ is the neighbourhood set of the neighbours of node *i*.

#### 2.1.2. Modularity

Modularity is an optimisation function that is used to evaluate the quality of a graph partition, which was designed by Newman and Girvan [5]. The larger the value of the modularity function, the better the quality of the detected communities [17,18]. The model is given in Equation (2).

(2) $$\;Q=\sum \;e_{ii}-a_{i}^{2}\;$$

$e_{ii}$ is the fraction of edges included in the community *i* and $a_{i}$ is the fraction of nodes’ degree included in the community *i*.

(3) $$\;e_{ii}=E_{i}\;/\;m\;$$

where $E_{i}$ is the number of edges contained inside the community *i* and *m* is the total number of edges in *G*.

(4) $$\;a^{2}=\frac{\sum_{v\in C_{i}}d_{v}}{\sum_{v\in G}d_{v}}\;$$

where $C_{i}$ is the community *i* and $d_{v}$ is the degree of node *v*.

#### 2.1.3. Betweeness Centrality

The Betweeness centrality of a node *v*, first designed by Freeman [35], is given in Equation (5):

(5) $$\;C_{B}\left(v\right)=\sum_{\begin{array}{c} s\neq v\neq t\\ s,v,t\in V \end{array}}\frac{\rho_{st}\left(v\right)}{\rho_{st}}\;$$

where $\rho_{st}\left(v\right)$ is the number of shortest paths from node *s* to node *t* that pass through node *v*, and $\rho_{st}$ is the number of shortest paths from node *s* to node *t*.

#### 2.1.4. Bridging Coefficient and Bridging Centrality

The Bridging coefficient is defined as

(6) $$\;BC\left(v\right)=\frac{d{\left(v\right)}^{-1}}{\sum_{i\in N(v)}\frac{1}{d(i)}}\;$$

where d(v) is the degree of node *v* and N(v) is the set of neighbours of node *v*. Bridging centrality, on the other hand, is used to quantitatively measure the extent of bridging capability of all nodes in a network. Comparatively to other components on the same network, the bridge-nodes are identified on the basis of their high value of bridging centrality [6,7]. The bridging centrality $C_{R}\left(v\right)$ of a node *v* is defined by

(7) $$\;C_{R}\left(v\right)=BC\left(v\right)\times C_{B}\left(v\right)\;$$

where BC(v) is the Bridging coefficient and $C_{B}\left(v\right)$ is the Betweeness centrality.

#### 2.1.5. Clustering Coefficient

Clustering coefficient measures the degree of clustering that exists between node *v* and its direct neighbours [6]. The model is given in Equation (8).

(8) $$\;Cl\left(v\right)=\frac{2L}{d_{v}\left(d_{v}-1\right)}\;$$

where $d_{v}$ is the degree of node *v* and *L* is the number of links between $d_{v}$ neighbours of node *v*.

### 2.2. The Algorithm

The steps involved in the implementation of the proposed method for detecting disjoint nodes, disjunct nodes and overlapping nodes are stated in Algorithm 1. First, assign the desired number of partitions *P* to be detected. Initialise modularity Q=0 and create a copy of the network $G^{{}^{\prime }}\leftarrow G$. Then, compute the bridging centrality value $C_{BR_{i}}$ of all nodes in the network *G*. Select the node $B_{r_{i}}$ with the highest bridging centrality value. Compute the node similarity values between $B_{r_{i}}$ and all of its neighbours. Select the nodes that return the least node similarity value and delete the links/edges connecting them to $B_{r_{i}}$. Repeat the cycle until the number of connected components, modules or partitions of $G^{{}^{\prime }}==P$. In other words, the algorithm loops and keeps count of the number of modules/partitions until the network is divided up into total number of desired partitions *P* which was assigned at the beginning of the experiment. Assign all partitions with components greater than 1 to cluster nodes $C_{cluster}$. Find all single node partitions SP and compute their clustering coefficient $Cl_{coeff}$ from the original network *G*. Classify SP as neutral node $C_{neutral}$ if $Cl_{coeff}=0$, or overlapping node $C_{overlap}$ otherwise. Compute the quality of the resultant communities’ modularity, *Q*, and display the cluster nodes $C_{cluster}$, neutral node $C_{neutral}$ and overlapping node $C_{overlap}$.
**Algorithm 1** Multi-type Node Detection Algorithm     **Input:** Network *G*; desired number of partitions *P*     **Output:**
$C_{cluster}$, $C_{neutral}$, $C_{overlap}$, *Q*1:**initialize**Q=0, **copy**$G^{{}^{\prime }}\leftarrow G$;2:**compute**$C_{BR_{i}}=bridgingcentrality\left(G^{{}^{\prime }}\right)$     ▹ use Equation (7);3:**select**$B_{r_{i}}\leftarrow \max\left(C_{BR_{i}}\right)$   ▹ nodes with max. bridging centrality value;4:$Neb_{Br_{i}}\leftarrow \mathrm{find}\;\left(neighbours\left(B_{r_{i}}\right)\right)$;5:**if**$Neb_{Br_{i}}\leq 1$**then**6:    continue;7:**end if**8:**compute**$sim(B_{r_{i}},Neb_{Br_{i}})$,     ▹ node similarity, use Equation (1);9:**find**$\min(sim\left(B_{r_{i}},Neb_{Br_{i}}\right))$      ▹ remove links;10:**repeat**11:    2–1112:**until** number_connected_components$\left(G^{{}^{\prime }}\right)$ == *P*13:$C_{cluster}==\mathrm{find}\left(connected\_components\left(G^{{}^{\prime }}\right)>1\right))$; $SP==\mathrm{find}(connected\_components\left(G^{{}^{\prime }}\right)==1))$;     ▹ SP refers to Single Node Partitions14:**compute**$Cl_{coeff}=clusteringcoeff\left(G,SP\right)$; **calculate***Q*;15:**if**$Cl_{coeff}=0$**then**16:    $C_{neutral}\leftarrow SP$17:**else**18:    $C_{overlap}\leftarrow SP$19:**end if**20:**print**$C_{cluster},C_{neutral},C_{overlap},Q$


### 2.3. Properties of an Isolated Bridge-Node

**Figure 1:**
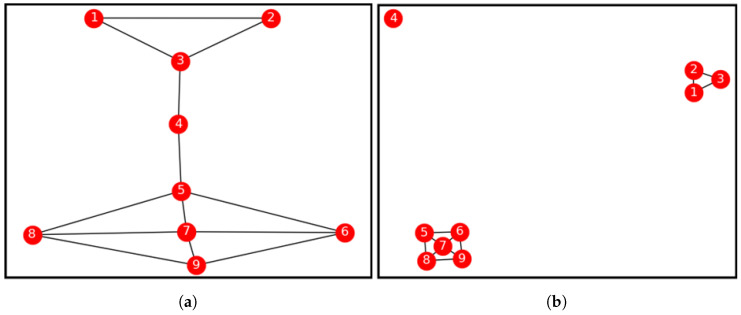
Example synthetic network. (**a**) Full network. (**b**) Fragmented network.

From the synthetic graph displayed in Figure 1a, we note that node $v_{4}$ has the highest bridging centrality value contained in Table 1. Further computations of the node similarity values between node $v_{4}$ and its neighbours nodes $v_{3}$ and $v_{5}$ returned the value 0, i.e., $sim\left(v_{4},v_{3}\right)=sim\left(v_{4},v_{5}\right)=0$. When the links connecting these nodes are disconnected, the network *G* disintegrates. This makes node $v_{4}$ become an isolated node as it has no similarity with any of its neighbours, yet it is very vital in bridging communities. From Table 2, we note that edges G(4,5);G(5,4) and G(4,3);G(3,4) returned the highest edge-betweeness values, respectively. These are the edges which link node $v_{4}$ with its neighbour’s nodes $v_{5}$ and $v_{3}$, respectively. Even though these edges have the highest edge-betweeness values, they are linked to an isolated bridge-node, which cannot form a community with any of its neighbours because it has zero node similarity values with them. The network *G* is disconnected into two distinct communities, with node $v_{4}$ not belonging to any particular community. Therefore, we designate node $v_{4}$ as a disjunct node without any community. This also demonstrates that, with respect to bridge-nodes, the link that yields the least node similarity value is same link with the highest edge-betweeness centrality value. In other words, node similarity has an inverse correlation with edge-betweeness centrality.

We can summarise the properties of an isolated-bridge node as follows.

- They are bridge-nodes.
- They have degree $k_{i}>1$.
- They have no path linking back to them. In other words, they do not share common nodes with any other node on the network. i.e., $|n_{i}\cap n_{j}|=\emptyset$. Therefore, they have zero node similarity values with all of their neighbours.

## 3. Results, Evaluation and Discussion

The algorithm is implemented with PYTHON3.7 and related packages (Networkx [36], Numpy [37,38], Matplotlib [39] and Scipy [40]) and run on a computer with Windows 7 OS (64-bits), Intel (R) Core${}^{\left(TM\right)}$ i7-4790 CPU (3.60 GHz) and 4 GB RAM.

### 3.1. Tests on Artificial Networks

**Figure 2:**
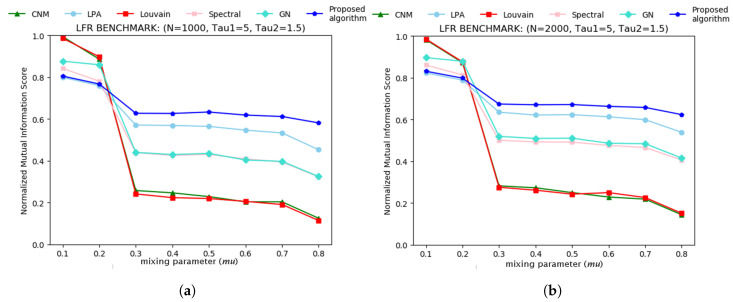
(**a**) Normalised mutual Information performance comparison of the proposed algorithm using Lancichinetti–Fortunato–Radicchi (LFR) benchmark. Number of nodes $N=1000,\tau_{1}=5,$$\tau_{2}=1.5,<k>\;=10,\min_{C}=20,\max_{C}=50$. (**b**) Normalised mutual information performance comparison of the proposed algorithm using LFR benchmark. Number of nodes $N=2000,\tau_{1}=5,$$\tau_{2}=1.5,<k>\;=10,\min_{C}=20,\max_{C}=60.$ The mixing parameter mu ranges from 0 to 0.8 with a step increment of 0.1.

The proposed algorithm was tested on Lancichinetti–Fortunato–Radicchi (LFR) benchmark [1,41] against the greedy algorithm of Clauset, Newman and Moore (CNM) [26]; Linear Propagation algorithm (LPA) [28]; Louvain algorithm (Louvain) [27]; Spectral Clustering algorithm (SPA) [29,30]; and Girvan-Newman algorithm (GN) [4]. The algorithm implemented in the work of Yuan et al. [19] was not included in any of the experiments in this work as we could not re-implement it. In the LFR benchmark, *N* is the number of nodes rendered in the network by the benchmark. $\tau_{1}$ and $\tau_{2}$ represent the power law exponent of the degree distribution and the power law exponent of the community size distribution produced in the network, respectively. <*k*> is the average degree of nodes in the network, and the mixing parameter μ is the fraction of intra-community links or edges connecting each node. $\min_{C}$ and $\max_{C}$ are the minimum size of communities and the maximum size of communities, respectively. The results obtained from the LFR benchmark, as shown in Figure 2a,b, indicate that the quality of communities detected by all the algorithms, except for the proposed algorithm deteriorates sharply at mixing parameter μ = 0.2. The proposed algorithm decline steadily in contrast to LPA, GN and SPA algorithms until μ = 0.3. The implication is that from μ≤0.3 qualities of communities detected are very good, but from μ>0.3, the qualities of the communities detected deteriorate. In any case, the proposed algorithm performs better than the other compared algorithms. For the LFR benchmark experiment in Figure 2a, we set N=1000 nodes, $\tau_{1}=5,\tau_{2}=1.5,<k>\;=10,\min_{C}=20,\max_{C}=50$. The number of communities to be detected was set at 100 for the proposed algorithm, GN and SPA. Likewise, In Figure 2b, we set N=2000 nodes, $\tau_{1}=5,\tau_{2}=1.5,<k>\;=10,\min_{C}=20,\max_{C}=60$. The number of communities to be detected was set at 200 for the proposed algorithm, GN and SPA. Due to the high CPU time in computing GN and the proposed algorithms, we did one iteration only.

### 3.2. Tests on Real-World Network Datasets

**Figure 3:**
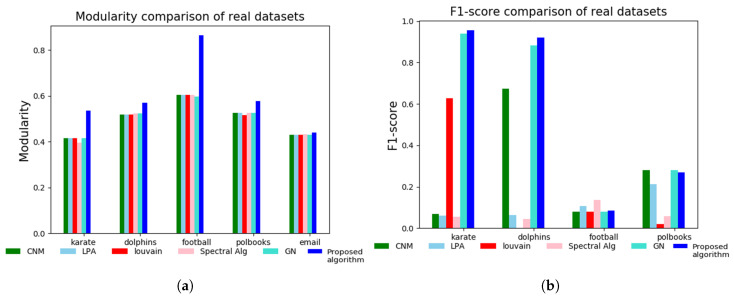
(**a**) Modularity measure comparison among CNM, LPA, Louvain, SPA, GN and the proposed algorithm. (**b**) F1-score comparison among CNM, LPA, Louvain, SPA, GN and the proposed algorithm. The email network is ommitted in the F1-score computation due to unavailability of its ground-truth data.

We further demonstrate the efficiency of the proposed algorithm with real-world datasets such as Zachary’s karate club network (Karate), Dolphins network (Dolphins), American football club network (Football), Kreb’s network of political books (Polbooks) and email data from European research institution (Email). Nodes and edges are indicated as *n* and *m*, respectively, whereas ground-truth represents the number of communities in the original network as shown in Table 3. The performance of the proposed algorithm is tested on real datasets against CNM, LPA, Louvain, SPA and GN algorithms using modularity measure and F1-score, which is an average of precision and recall computed from ground-truth community dataset and detected community dataset [32]. For modularity measure comparison among the stated algorithms, the number of communities to be detected for karate club network was set at 3 for SPA, GN and the proposed algorithm. For the dolphins network, the number of communities to be detected was set at 4 for SPA, GN and the proposed algorithm. For football network, the number of communities was set at 12 for SPA and GN. The proposed algorithm detected at most nine communities in the football network. Therefore, the number of communities was set at 9. For the polbooks network, the number of communities were set at 4 for SPA, GN and the proposed algorithm. For the email network, the number of communities was set at 42 for SPA and GN. Just like in the case of football network, the proposed algorithm detected at most 30 communities in the email network. Therefore, the number of communities was set at 30. As shown in Figure 3a, the proposed algorithm outperformed the compared algorithms in karate club network, dolphins network, football network and polbooks network. In the email network, the proposed algorithm performed marginally above the other algorithms. In Figure 3b, the proposed algorithm performed better than the other algorithms in Karate network and Dolphins network. Expectedly, LPA and Spectral algorithms performed better ahead of the proposed algorithm, CNM, Louvain and GN algorithms in the football network. This could be as a result of the proposed algorithm detecting at most nine communities in this network. In the polbooks network, the performance of the proposed algorithm is good but less than the performance of CNM and GN algorithms. The email network was not considered for the F1-score computation due to unavailability of its ground-truth dataset.

#### 3.2.1. Zachary’s Karate Club Network

**Figure 4:**
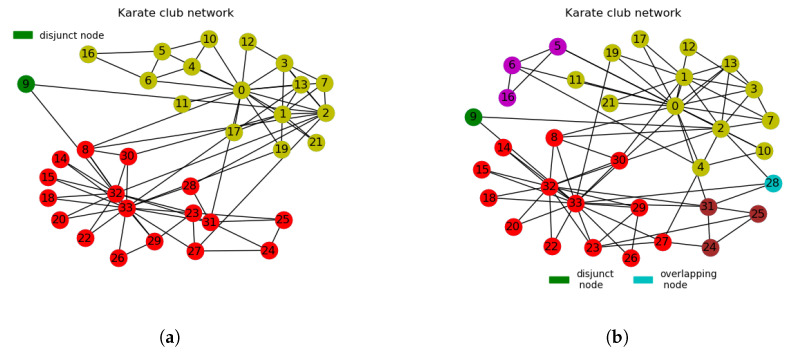
(**a**) Zachary’s karate club network partitioned into 2 communities with 1 disjunct node. (**b**) Zachary’s karate club network partitioned into 4 communities with 1 disjunct node and 1 overlapping node. The partitions overlapped by node 28 are overlapping communities. The rest of the nodes not indicated on the legends in Figure 4a,b represent different communities according to their respective colours.

The results obtained show that the proposed algorithm is quite efficient in identifying disjoint nodes, disjunct nodes and overlapping nodes. In Zachary’s karate club network, shown in Figure 4a, the proposed algorithm detected three partitions (two cluster node partitions and one single node partition). The two cluster node partitions (disjoint nodes) are the two main communities whereas the single node partition (node 9) is a disjunct node. The ground-truth community of this network comprises two main partitions, as indicated in Table 3, but some useful clusters can be found at sub-modular levels as indicated in Figure 4b. The proposed algorithm was able to recover the metadata of node 9 as a disjunct node. This corresponds to what is reported in the work of Peel et al. [24], where person number 9 is indicated to likely have possessed neutral political inclination neither towards the karate club president nor the club instructor during the feud between these two persons that eventually resulted in the split of the karate club into two. Often, most algorithms fail to recover this particular node or they mislabel it [24]. In Figure 4b, the proposed algorithm detected four main communities with one disjunct node (node 9) and one overlapping node (node 28). The partitions overlapped by node 28 are overlapping communities. Information revealed at sub-modular levels of partitions can be very useful in situations where one needs to examine the connections and relationships among nodes at sub-modular structures. Node 9 (displayed in green) in Figure 4a,b and node 28 (displayed in cyan) in Figure 4b are shown as being isolated, but a careful examination shows that only node 9 meets the requirements to be classified as a disjunct node. Node 28 is an overlapping node as it has at least an edge linking back to it and it shares clusters with two of its neighbours (nodes 31 and 33), which are in different communities that form the overlapping communities. Yuan et al. [19] correctly classified this node as an overlapping node which corresponds to node 29 in their work. Also, the proposed algorithm achieved modularity value of 0.5789 at three communities as indicated in Table 4, which is greater than SPA and GN’s modularity values of 0.4188 and 0.4188 respectively at three communities each. CNM and LPA returned three communities each with modularity values of 0.4198 and 0.4198, respectively. At 4 communities, the proposed algorithm achieved modularity value of 0.5940 which is greater than the modularity value of 0.4156 achieved by Louvain algorithm at four communities. It is very apparent that the modularity values achieved by the proposed algorithm on the Karate club network are higher than those of the other algorithms considered for comparison as can be seen in Table 4. This is a clear indication that the proposed algorithm attains better clustering quality than the compared algorithms.

#### 3.2.2. Dolphins Network

**Figure 5:**
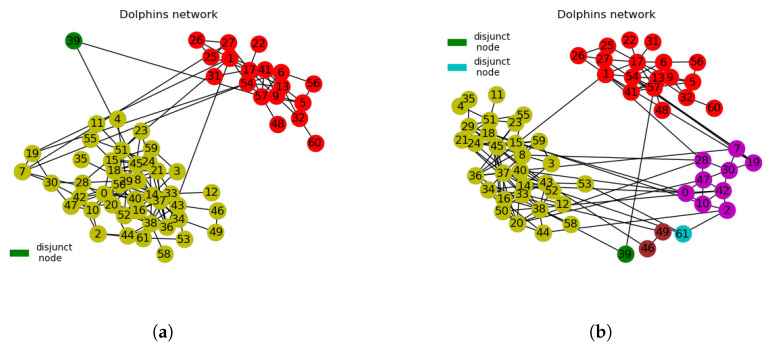
(**a**) Dolphins network partitioned into 2 communities with 1 disjunct node. (**b**) Dolphins network partitioned into 4 communities with 2 disjunct nodes. The rest of the nodes not indicated on the legends in Figure 5a,b represent different communities according to their respective colours.

The proposed algorithm can choose the number of partitions to be returned. This way, modular structures at lower hierarchies are revealed. In the dolphins network, shown in Figure 5a, the two larger communities (disjoint nodes) are clearly indicated with one disjunct node (node 39). Yuan et al. [19] reported node 40, which corresponds to node 39 in our work, as an overlapping node rather than as a disjunct node, but we understand that this is as a result of differences in methods implemented in the respective algorithms. The proposed algorithm achieved modularity value of 0.6989 at four communities, which is higher than the modularity values of 0.5188 for CNM and SPA each and 0.4156 for GN at four communities. LPA and Louvain achieved modularity values of 0.5196 and 0.5268, respectively, at six communities each. These values are less than the modularity value of 0.6989 achieved by the proposed algorithm as indicated in Table 4.

#### 3.2.3. The Other Networks

**Figure 6:**
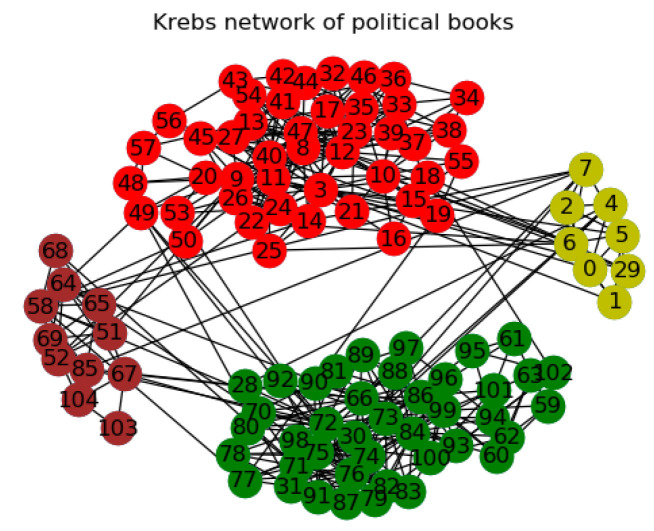
Kreb’s network of political books at 4 communities.

In Kreb’s network of political books, the proposed algorithm achieved modularity value of 0.5905 at four communities (all disjoint nodes) in comparison to CNM, Louvain, SPA and GN’s modularity values of 0.5266, 0.5270, 0.5270 and 0.5266, respectively, at four communities each. At 8 communities, LPA algorithm achieved modularity value of 0.5268 as against the proposed algorithm’s modularity value of 0.6964 at eight communities. Yuan et al. [19] classified nodes 30 and 86 as overlapping nodes at four communities. Our results show that these nodes which correspond to nodes 29 and 85 in our work as shown in Figure 6 are members of clusters.

In American college football network, the proposed algorithm could detect at most nine communities, contrary to the ground-truth of 12 communities indicated in Table 3 and what others reported in the literature. The quality of the communities detected by the proposed algorithm is still quite high in comparison to other methods with modularity value of 0.8641. We noticed that six of the conferences combined to form three bigger conferences. Clauset et al. [26] reportedly detected six communities with modularity value of 0.6046. Yuan et al. [19] reportedly detected 10 communities with node 37 as overlapping node, whereas the proposed algorithm does not have any overlapping node.

In the email data network of European research institution, the proposed algorithm detected at most 30 communities with modularity value of 0.4415. CNM algorithm returned 44 communities with modularity value of 0.4324. LPA algorithm returned 38 communities with modularity value of 0.4306. Louvain algorithm returned 28 communities with modularity value of 0.4322. SPA and GN algorithms’ number of communities were fixed at 42 each and they achieved modularity values of 0.4314 and 0.4328, respectively. These values are presented in Table 4.

The method developed in this paper leads the way in multi-type node detection on networks contrary to previous methods that detect either only cluster nodes or overlapping nodes. Most of the methods often rarely identify disjunct nodes, which are integral parts of complex networks that play various significant roles. We further highlighted the unique properties of disjunct nodes which prior to this time had not been properly characterised by any other work. From our observation, the disjunct nodes can have several connections to their direct neighbours but when the network is disintegrated, they are shown to be isolated. In other words, they do not belong to any community. Discovery of these types of nodes could be very useful in certain instances to determine the actual impact they may have on the network and their neighbours. For example, a protein molecule in a network of protein–protein interactions (PPI) can connect other modular protein clusters and could be revealed to be a disjunct protein molecule at a sub-modular level when the network is divided up. One can investigate the significant roles such protein molecules play and the possible effects their malfunction can have on the surrounding protein molecule clusters. With an understanding of something of this nature, careful study of biological cells can help in designing drugs for disease treatment and epidemic controls. In computer networks, this can be very helpful in the design of network configuration of computers. Also, in the fight against drugs and related crimes, a drug mule or courier who works for drug cartels, but is not necessarily a member of any of the drug cartels, can be intercepted and the cartels infiltrated. Another possible area of interest might be in the design of power grid networks.

To actualise our set objectives, we used the bridging centrality metric [6] as a tool to help us determine bridge-nodes. We also used the Jaccard similarity coefficient to help determine the level of similarity or relationship between the bridge-nodes and their neighbours. This helped us to distinctly identify and classify the node types. A clear distinction was made between the disjunct nodes and the overlapping nodes. It is imperative we point out that our method and objectives are quite different from the method and objectives in [7]. Hwang et al. [7] proposed bridge-cut algorithm which is based on bridging centrality of edges. We have not compared the performance of these two methods as it’s not part of the scope of this present work.

Additionally, we set the number of desired output partitions ahead of time before executing this algorithm. This allows one to adjust the number of partitions to be returned so as to ensure careful study of the multi-level hierarchical structures in networks. Such information as this can be very useful in disease control by deletion of certain edges connected to isolated or overlapping nodes. Some existing studies also support this point of view [7,48]. Differentiating multi-type nodes in a natural way on networks can equally be helpful in critical examination of cell interactions and drug designs, protein–protein networks, etc. [6,7]. It can also give insight to future studies and understanding of terrorist cells operations, illegal transfer of funds among terrorists, drug courier networks, organised crime gangs, power grids, internet infrastructure designs, road network designs and so on.

#### 3.2.4. Computational Complexity Analysis

The bridging centrality metric is bounded by the time complexity of betweeness centrality based on Brande’s betweeness algorithm, which is what is implemented in the Networkx python package used in this work. It is calculated in O(nm) time, where *n* and *m* are the total number of nodes and edges on a network, respectively [7,49]. Its space complexity takes $\Theta (n^{2})$ to be computed. The bridging coefficient consumes approximately $O(n{\left(\log n\right)}^{2})$ time [7]. The Jaccard similarity coefficient takes $O(m^{2})$ time to be computed [50]. Due to the recomputation of bridging centrality and Jaccard similarity coefficient after every iteration; therefore, our algorithm can be computed in a total time and space complexity of $O{\left(\left(nm\right)+\left(m^{2}\right)\right)}^{2}$ and $\Theta (n^{2})$, respectively. The processing time expended on executing each algorithm on different networks is give in Table 5. The proposed algorithm only performs better than GN with respect to small networks and performs poorly in large networks.

#### 3.2.5. Limitations and Future Works

In future works, we hope to design an autonomous divisive algorithm that needs no parameters to stop the iteration. We also hope to make the algorithm scalable for very large networks because the betweeness centrality metric, as a global metric, has a high computational efficiency as indicated from the processing time in Table 5. This algorithm will be deployed in various application domains to explore further studies in these areas.

## 4. Conclusions

We designed a new algorithm that distinctly identifies and classifies multi-type nodes in network communities. Bridging centrality metric was used to calculate and select nodes with the highest bridging centrality value. Jaccard similarity coefficient was used to determine the level of similarity or relationship between the bridge-nodes and all of their neighbours. The nodes with the least similarity value were disconnected iteratively after which the bridging centrality of all nodes are recomputed until the stopping condition was met. We also validated the existence of disjunct/neutral nodes and highlighted the properties that characterise them. The results from extensive experiments done with real-world datasets show that this algorithm is efficient in distinctly discovering and classifying disjoint nodes, overlapping nodes and disjunct nodes, which are shown to be neutral nodes in terms of community membership. These results demonstrate the effectiveness of the proposed method and we believe that it will be of significant use in various application domains of community detection as well as arouse interests in future designs of an all inclusive community detection algorithms. This way, node connectivity relations can be revealed and studied better at sub-modular levels of different complex systems.

## Figures and Tables

**Table 1:** Bridging centrality and node similarity values of nodes in network *G*.

Iteration Count	Node ID.	Bridge Centrality Value	Neighbours.	Node Similarity Value
1st	4	0.4592	3	0
			5	0
2nd	7	0.0045	5	0.2857
			6	0.2857
			8	0.2857
			9	0.2857

**Table 2:** Edge-Betweeness values of links/edges with the highest values in network *G*.

Edge	Edge-Betweeness Value
G(4,5);G(5,4)	0.2778
G(4,3);G(3,4)	0.2500
G(1,3);G(3,1)	0.0972
G(2,3);G(3,2)	0.0972

**Table 3:** Properties and description of network datasets used.

Network	n/m	Ground-Truth	Description	Ref
Karate Club	34/78	2	Friendship network of karate club members	[42]
Dolphin	62/159	2	Association network of bottlenose dolphins	[43]
Polbooks	105/441	3	A co-purchasing network of political books	[44]
Football	115/613	12	A game-scheduling network of teams	[45]
Email EU	1005/16706	42	European research institution’s email data	[46,47]

**Table 4:** Modularity values and number of communities gotten from real complex networks. Number of communities indicated against CNM, LPA and Louvain are auto-generated since they do not need prior parameters before execution. The proposed algorithm could detect at most 9 communities for the football network and 30 communities for the Email network. The modularity values shown against SPA, GN and the proposed algorithms for Karate, Dolphin and Polbooks networks are based on the smallest number of communities returned among CNM, LPA and Louvain algorithms.

Modularity *Q* and Number of Communities (C)						
**Network**	**CNM**	**LPA**	**Louvain**	**SPA**	**GN**	**Proposed Algorithm**
**Karate**	0.4198 C=3	0.4198 C=3	0.4156 C=4	0.4188 C=3	0.4188 C=3	0.5789 C=3
**Dolphin**	0.5188 C=4	0.5196 C=6	0.5268 C=6	0.5188 C=4	0.4156 C=4	0.6989 C=4
**Polbooks**	0.5266 C=4	0.5268 C=8	0.5270 C=4	0.5270 C=4	0.5266 C=4	0.5905 C=4
**Football**	0.6046 C=6	0.6043 C=11	0.6044 C=10	0.6046 C=12	0.6043 C=12	0.8641 C=9
**Email**	0.4324 C=44	0.4306 C=38	0.4322 C=28	0.4314 C=42	0.4328 C=42	0.4415 C=30

**Table 5:** CPU execution time of the algorithms in seconds.

Network	CNM	LPA	Louvain	SPA	GN	Proposed Algorithm
**Karate**	0.0037	0.0012	0.0110	0.0110	0.0467	0.0311
**Dolphin**	0.0147	0.0101	0.0301	0.0604	0.1264	0.0960
**Polbooks**	0.0232	0.0061	0.0400	0.0712	1.3444	0.8653
**Football**	0.0513	0.0290	0.0655	0.1937	5.5780	3.5012
**Email EU**	2.3331	0.1486	1.3961	1.4739	324.79	6804.38
